# Human liver flukes in China and ASEAN: Time to fight together

**DOI:** 10.1371/journal.pntd.0007214

**Published:** 2019-04-25

**Authors:** Men-Bao Qian, Xiao-Nong Zhou

**Affiliations:** 1 National Institute of Parasitic Diseases, Chinese Center for Disease Control and Prevention, Shanghai, China; 2 Key Laboratory of Parasite and Vector Biology, Ministry of Health, Shanghai, China; 3 Chinese Center for Tropical Diseases Research, Shanghai, China; 4 WHO Collaborating Center for Tropical Diseases, Shanghai, China; Seoul National University College of Medicine, REPUBLIC OF KOREA

The second forum on health cooperation between China and the Association of Southeast Asian Nations (ASEAN) was held in Guangxi, China, on September 19 and 22, 2018 [1]. Important resolutions, including those on the cooperation between China and ASEAN member countries on traditional medicine, management of hospitals, youth exchanges, and the prevention and control of diseases, were proposed at the regional forum [1]. This regional cooperation provides a platform and a potential opportunity to fight the menace of human liver flukes together. Two human liver flukes—namely, *Clonorchis sinensis* and *Opisthorchis viverrini*—exert a huge burden in China and Southeast Asia [2, 3]. Infections due to these human liver flukes are predominantly endemic in China and several ASEAN member countries as a result of the traditional and rooted dietary habit of ingesting raw freshwater fish. There are about 15 million people infected with *C*. *sinensis* globally, out of which 13 million are domiciled in China and 1 million in northern Vietnam [2]. *O*. *viverrini* infection accounts for about 8.6 million cases, with distribution in the following ASEAN member countries: Thailand (6 million cases), Laos (2 million cases), and Cambodia (0.6 million cases) [3]. *O*. *viverrini* infection is also endemic in southern Vietnam. Recent findings have revealed the distribution of *O*. *viverrini* infection in Myanmar, which has given us more insight on human liver fluke distribution in the region [4]. Thus, China and ASEAN member countries share more than 90% of the clonorchiasis burden and about 100% of the opisthorchiasis viverrini burden. Both infections pose significant health concerns in the region. Long and chronic infections could lead to high morbidity in liver and the biliary system (e.g., gallstone, cholecystitis, and cholangitis) [2, 5, 6]. In particular, only three parasites are definite carcinogens, including both *C*. *sinensis* and *O*. *viverrini* [7], which are responsible for a conservative estimate of 7,000 new cases of cholangiocarcinoma yearly [8]. Thus, China and ASEAN member countries are highly afflicted with cholangiocarcinoma [9].

In spite of the persistent high burden, human liver flukes are yet to receive adequate attention compared with other neglected tropical diseases, and this enhances the disease persistence in the region. For example, Guangxi, where the health cooperation meeting was held, is highly endemic with clonorchiasis. Most neglected tropical diseases have been effectively controlled in Guangxi, especially lymphatic filariasis, which was eliminated in 1985 [10], and schistosomiasis transmission, which was interrupted in 1988 [11]. However, there has been an increasingly widespread distribution and prevalence of clonorchiasis over the past decades [12, 13].

A basis for the potential cooperation between China and ASEAN on human liver flukes already exists. First, *C*. *sinensis* and *O*. *viverrini* belong to the same family, and thus they have many similarities in biology, pathobiology, carcinogenicity, epidemiological determinants, diagnosis, treatment, control, and prevention [2, 3]. Second, research and development on liver flukes have already achieved some breakthroughs in different countries—e.g., mapping and surveillance in China, drug development in China and Laos, the carcinogenic mechanism, diagnosis and management of cholangiocarcinoma in Thailand, and the management of aquaculture in Vietnam [14–19]. All of these efforts provide a basis for future cooperation. Third, there exist good cooperative platforms, such as the Regional Network for Asian Schistosomiasis and other Helminth Zoonoses (RNAS^+^), of which all countries endemic for clonorchiasis and opisthorchiasis viverrini are members [20]. Numerous training courses, which have attracted more than 400 participants, have been held through this network since 2000, and several collaborative projects have been completed through RNAS^+^, some of which are still ongoing.

The objective of fighting liver flukes via cooperation could be achieved through diverse approaches such as talent development; joint research and development of tools, products, and strategies; and the establishment of cooperative pilot projects. The health cooperation forum provides a potential opportunity for cooperation by targeting the promotion of effective and sustainable control of liver flukes in the region [2, 21–24].

Some special objectives that require attention are as follows:

1. To construct the regional database. Accurate endemic maps for human liver flukes in the region are yet to be available, and this hinders control activities. This should not be limited to prevalence data; data on ecological and socioeconomic factors should also be included.
2. To estimate the disease burden. A full evaluation of disease burden should contain both health loss and economic cost. Regarding health loss, fatal cholangiocarcinoma, chronic liver and biliary conditions, and early nonspecific symptoms need to be evaluated comprehensively. Economic burden should include direct and indirect costs related to hospitalization, loss of productivity due to disability, premature mortality, etc.
3. To explore the determinants. Cultural, psychological, and behavioral factors contribute to the persistence of liver flukes in endemic areas. Providing insights on these determinants would greatly benefit the design of effective measures and sustainable strategies. More importantly, attitudinal change by doing away with eating raw freshwater fish will facilitate primary prevention of liver fluke infections, and this is key for long-term and sustainable control, which could eventually lead to disease elimination as a public health problem.
4. To develop screening and diagnostic tools. Rapid and simple screening tools are required to detect endemic communities and individuals at risk. Sensitive and specific serologic tests are needed in clinical settings. Rapid diagnostic tests, such as dipsticks, are important for field tests. Additionally, field-friendly molecular diagnostic methods need to be explored, and they should have the ability to differentiate among different species of human liver flukes as well as between human liver flukes and other minute intestinal flukes.
5. To develop and evaluate new drugs. As an alternative to praziquantel, albendazole efficacy needs to be evaluated further, and the regimens should be optimized. Previous studies have shown the promising efficacy and safety of tribendimidine against both *C*. *sinensis* and *O*. *viverrini*. Further trials are expected to verify and optimize its usage before application.
6. To study the potential spread of the two liver flukes. The two flukes are usually believed to have separate preferences for transmission sites in endemic areas because of the different distributions of their respective first intermediate hosts. However, the potential to spread to opposite endemic areas and other nonendemic areas also requires attention and should be studied, especially considering global climate change.
7. To strengthen modeling application. Studies using the application of modeling techniques are grossly inadequate for human liver flukes. These techniques help illustrate transmission dynamics, predict risk maps, design control measures, determine the cost-effectiveness, etc.
8. To explore new control measures. The strategies, effectiveness, cost, and sustainability of different measures including chemotherapy; information, education, and communication techniques; control of animal reservoirs; establishment of sanitation; management of aquaculture; and the integration of diverse measures should be investigated and evaluated.

The necessity, urgency, and feasibility of controlling human liver flukes through technical cooperation could be demonstrated to the governments in endemic countries, and this would promote its potential inclusion in national health policy. Then, multisectoral cooperation could be established between health departments and other departments, especially education and agriculture, which will guarantee its effectiveness and sustainability. We therefore expect that human liver flukes will be effectively tackled through our cooperation mechanism, and this will corroborate the achievements of other cooperation in this region.
